# Alphabetism in reading science

**DOI:** 10.3389/fpsyg.2014.00752

**Published:** 2014-07-18

**Authors:** David L. Share

**Affiliations:** Department of Learning Disabilities, Faculty of Education, University of HaifaHaifa, Israel

**Keywords:** learning to read, reading, anglocentrism, alphabetism, language, writing systems, orthography

There has been mounting concern among social scientists that conclusions from studies conducted on highly educated populations from affluent European cultures may have limited applicability to human behavior in general (Henrich et al., 2010). Similar reservations have also been voiced in the fields of language (Evans and Levinson, 2009) and literacy (Share, 2008a; Frost, 2012). Reading research, in particular, has been overwhelmingly dominated by work on English, which appears to be an outlier among European alphabets (Seymour et al., 2003; Share, 2008a). I have argued that because spelling–sound relations are so complex in English orthography, much of reading research has been confined to a narrow Anglocentric research agenda addressing theoretical and applied issues with only limited relevance for a universal science of reading and literacy.

My intention here is not to reiterate my 2008 arguments or even expand them, but to move on to another major obstacle to progress. Before moving on, however, I would like to add a note of optimism to the Anglocentrism debate. In recent years, interest in other languages has indeed begun to emerge from the shadows probably because the scientific community of Anglo-American reading researchers has felt itself “come of age” as a substantial body of well-replicated and converging findings has coalesced in recent years, at least on several key topics such as word identification and dyslexia (Vellutino et al., 2004; Snowling and Hulme, 2005; DeHaene, 2009; Rayner et al., 2012). The field is now witnessing important first steps toward universal models of reading (Perfetti, 2003; Perfetti et al., 2005; Ziegler and Goswami, 2005; Frost, 2012) as well as a growing number of linguistically and grammatologically informed studies emerging outside the confines of English and other European alphabets (Nag and Perfetti, 2014; Saiegh-Haddad and Joshi, 2014; Verhoeven and Perfetti, 2014). It is still the case, nonetheless, that the theoretical and applied frameworks developed for English are all too frequently being applied to other languages and writing systems without due consideration for linguistic and writing system diversity. Almost all publications by English-language researchers continue to omit any “…*in English*” qualification in the titles of their papers—“*A New Whiz-Bang+++ Model of Learning to Read*”… *in English*?—as if the results of studies conducted in English alone enjoy the privileged status of universal applicability, unlike researchers investigating other languages who are obliged to qualify their findings by adding the “…*in Chinese/Arabic/Korean* etc.” disclaimer which automatically demarcates the findings as language-specific and hence not necessarily universally applicable.

Here, I focus on yet another “-ism,” which I call “alphabetism”; the belief that alphabetic writing systems are inherently superior to non-alphabetic systems, and which, like Anglocentrism, has also stymied psychologists' and educators' thinking about learning to read across diverse writing systems. Here too, I join other scholars who have also expressed concerns about “alphabetolatry,” or alphabetic “supremacism” (e.g., Rogers, 1995). Looking around the globe, it is apparent that most individuals do not acquire literacy in a European alphabet, yet in many parts of the (non-European) world, the belief that alphabetic orthographies are the ideal has led to calls to alphabetize or discard non-alphabetic scripts. Needless to say, these proposals have profound ramifications for instruction and curriculum.

In the past, many influential Western scholars explicitly argued that alphabets are inherently superior to non-alphabetic writing systems (Taylor, 1883; Gelb, 1963; Havelock, 1982). The shelves of most college libraries abound with volumes whose very titles idealize the alphabet (e.g., Diringer's *The Alphabet: A Key to the History of Mankind;* Moorhouse's *Triumph of the Alphabet*). When reading researchers today seek enlightenment on the subject of writing systems they refer to Gelb—the founding father of the field of “grammatology” (Gelb, 1963). Like Taylor (1883) before him, Gelb (1963) propounded an evolutionary view of writing system history from “primitive” pre-alphabetic systems to alphabetic. Consistent with the “ontogeny recapitulates phylogeny” idea, Gelb's inexorable “*three great steps [logographic-to-syllabic-to-alphabetic] by which writing evolved from the primitive stages to a full alphabet*” (p. 203) was embraced by almost all reading researchers, despite its repudiation by subsequent scholarship in the field of writing systems research (Mattingly, 1985; Olson, 1989; Daniels, 1992, in press; Rogers, 2005; Coulmas, 2009). Foremost among these, perhaps, was Ferreiro in her Piagetian classic *Literacy before Schooling* (Ferreiro and Teberosky, 1979) and, subsequently, a series of stage-oriented theories of reading and writing development (Piagetian and non-Piagetian alike) all referring to pre-alphabetic and alphabetic stages (Gough and Hillinger, 1980; Marsh et al., 1981; Frith, 1985; Ehri, 2005). It needs to be pointed out, however, that the “culture” of alphabetism, like culture in general, is often “invisible”; its presence more often discernible in acts of omission rather than commission. Nonetheless, this alphabetic bias is ubiquitous and is manifest in;

1. Unqualified generalizations about reading “across languages” and/or “across orthographies” in papers that refer almost exclusively to English or to European alphabets (Ziegler and Goswami, 2005; Goswami, 2010; Ziegler et al., 2010; Caravolas et al., 2013; Ehri, 2014).
2. Implicit or explicit acceptance of Gelb's long-discarded evolutionary theory in leading texts on reading development aimed at educators,… “*Taking the final step toward the creation of a true alphabetic writing system, the Greeks assigned a symbol to each consonant and vowel of their language… In many ways, the individual development of the children who are discovering the alphabetic principle in English writing recapitulates human history*,” (Moats, 2000, pp. 82–83).
3. Even the most up-to-date and authoritative texts on the psychology of reading (e.g., DeHaene, 2009; Rayner et al., 2012) continue to regurgitate Gelb's views.

*“[I]n an evolutionary sense, the alphabet is the “fittest…” p. 37”; The history of writing suggests a clear evolutionary trend…These systems evolved to a logographic system, which in turn evolved to syllabic systems and finally to alphabetic systems…Such an evolutionary argument suggests that alphabets are fitter (in the Darwinian sense*)… Rayner et al. (2012, pp. 46–47).

4. Reference to non-alphabetic systems as imperfect or defective (e.g., Hannas, 2003; Rayner et al., 2012) as well as attempts to reframe non-alphabetic systems such as the Brahmi-derived Indic (abugidic/aksharik) scripts as alphabetic (Rimzhim et al., 2014).

*…“The Semitic writing systems…and the languages of India still incompletely represent vowels. p 36… In this sense, many of these scripts are not fully alphabetic.”* Rayner et al. p. 37.

*“The Phonecian system, however, was not perfect. It failed to represent all vowels… It was the Greeks who finally created the alphabet as we know it… For the first time in the history of mankind, the alphabet allowed the Greeks to have a complete graphic inventory of their language sounds.”* (DeHaene, 2009, p. 193).

*“The basic difference between Western alphabetic and East Asian syllabic writing acts on several levels to promote or inhibit creativity, particularly that associated with breakthroughs in science… syllabic literacy entails a diminished propensity for abstract and analytical thought… Certain Asian characteristics credited with blocking creativity, such as conservative political and social institutions and group-oriented behavior, derive in part from effects that the orthography has had on the minds of individuals,”* (Hannas, 2003, p. 203).

5. The use of alphabetic terminology (e.g., letters, graphemes) to describe and label the functional architecture (and even the anatomical brain structures) of reading (“letter detectors,” “letterbox area,” “universal letter shapes,” DeHaene, 2009) purported to be universal in reading. Whereas the concept of a letter (or grapheme) is widely used (but not entirely unproblematic) in European alphabets, it has questionable applicability to many writing systems such as Chinese characters, Japanese Kanji, Brahmi-derived Indic aksharas or Mayan glyphs. It has even been suggested that the notion of the “phoneme” as the fundamental unit of analysis of speech may be an artifact of West European alphabetic literacy (Daniels, in press).

Although some initial thoughts have been offered as to when an alphabet may or may not be the appropriate orthography (e.g., Perfetti and Harris, 2013), this topic is new to the agenda of reading science. Some historical background on the alphabet provides a valuable perspective on this issue.

## Some historical background

Contrary to popular belief, the alphabet did not originate among Semitic speakers, or their Egyptian neighbors, but was a uniquely Greek creation invented only once, and probably on the basis of a fortuitous misunderstanding of Phoenician writing (Daniels, 1992). An alphabetic writing system, with full and equal representation of consonants and vowels, was ideally suited to the unique features of Indo-European languages (Diringer, 1948; Taylor, 1883). It added vowel notation to the Phoenician abjad, which was also a segmental/phonemic system but represented (and only needed to represent) consonants alone. Would an alphabet ever have been needed had there been no Indo-European languages in the world? Indo-European languages have a large inventory of complex syllable structures, far too many for a syllabary such as Japanese. And because vowels are essential constituents of root morphemes (bat/bet/bit/but/beet/bite… etc.) the Semitic abjad would have been inadequate.

This uniquely European mutation was first disseminated throughout Europe with the spread of Christianity, then across the globe by European colonizers, traders, and, above all, missionaries who never thought to question whether their own writing systems would be optimal for non-European languages. They took it for granted that the ideal orthography was alphabetic, operating on the principle of one letter for one sound (phoneme) for both consonants and vowels under the motto “consonants as in English, vowels as in Italian.” (Gleason, 1996).

But are alphabets optimal? Well, we really don't know. There is, however, evidence suggesting that it cannot be assumed that alphabets are inherently superior and therefore the default choice of script. There are at least four lines of counterevidence converging on the conclusion that syllable-based writing systems are, in many cases, superior to alphabets.

1. Psychoacoustically, syllables are more “real” than phonemes (Liberman et al., 1967). Data from illiterates (Morais et al., 1987), pre-literates (Liberman et al., 1974), or persons literate in purely morpho-syllabic or syllabic systems (Read et al., 1986) confirm that syllables are easier to deal with than phonemes.
2. Historically, syllabaries appeared earlier and more often in ancient times (Rogers, 2005; Gnanadesikan, 2009), whereas the alphabet was a relative latecomer in the history of writing and appeared only once (Daniels, 1992). All new writing systems invented by non-literates who know that writing exists are syllabaries (Daniels, 1992).
3. Anthropologists have reported widespread literacy among indigenous peoples using syllabic systems in North America (among the Cree, McCarthy, 1995; Cherokee, Walker, 1969); Africa, (Scribner and Cole, 1981); and the Philippines (the Hanuno'o, Kuipers and McDermott, 1996).
4. Quasi-experimental studies suggest that young children are able to learn to read syllabically (abugidically) more easily than phonemically/alphabetically (e.g., Gleitman and Rozin, 1973; Asfaha et al., 2009). Asfaha et al., for example, investigated reading acquisition in four Eritrean languages that use either syllabic (abugidic) (CV) Geèz (Tigrinya and Tigre) or alphabetic Latin-based scripts (Kunama and Saho). Instruction in alphabetic Saho focuses on CV units, whereas alphabetic Kunama is taught alphabetically, i.e., phonemically. Asfaha et al. found that first graders learned to read the non-alphabetic Ge'ez far more easily than the alphabetic scripts in spite of the larger number of signs. Moreover, the abugidic CV-level teaching of alphabetic Saho produced superior results compared to alphabetic teaching of (alphabetic) Kunama^1^. There are also studies showing that children and adults who have struggled to learn to read alphabetically find it easier to learn to read a syllable-based orthography than an alphabetic orthography (Gleitman and Rozin, 1973; Moore et al., 2014).

My aim here is not to show that syllabic writing systems are superior to alphabetic systems, but simply that alphabets cannot be assumed a priori to be inherently superior to other writing systems. The crucial question (as discussed by Perfetti and Harris, 2013) is the match between language structure and writing system, in particular the size and complexity of the syllable inventory.

This issue leads to the more general question, What makes an orthography more or less optimal?

## Writing system efficiency and a universal model of learning to read

An efficient writing system must do two things simultaneously: represent sound and meaning (Rogers, 1995; Share, 2008b; Frost, 2012). This is no simple task, because these two aspects of writing must often be traded off against each other. I have termed these two dimensions of orthography *decipherability* and *automatizability*/*unitizability* (Share, 2008b). Orthographies can be regarded as dual-purpose devices serving the distinct needs of novices and experts (see Share, 2008a). Because all words are initially unfamiliar, the reader needs a means of deciphering new letter strings unassisted (see Share, 1995, 2008b, for more detailed discussion, and Ziegler et al., 2014 for an explicit computational instantiation of this notion). Here, the representation of recombinant sub-lexical phonological elements (either syllabic, sub-syllabic, or phonemic) is fundamental if a script is destined to be decipherable and learnable (Mattingly, 1985; Unger and DeFrancis, 1995). But the essence of skilled reading (as is the case with all human skills) is speed and effortlessness. To achieve fluent, automatized reading, the expert-to-be requires unique word-specific or morpheme-specific letter configurations that can be “unitized” and automatized for instant access to units of meaning. Here morpheme-level (and probably also word-level) representation is essential^2^. Both morpheme *distinctiveness* (<rite/right>) as well as morpheme *constancy* (<soft/soften>) are crucial for rapid silent reading (Rogers, 1995).

The corollary to this orthographic dualism is what goes on inside the reader's head. Initially unfamiliar words and morphemes become familiar units, as the novice reader's orthographic lexicon begins to grow. This “unfamiliar-to-familiar” or “novice-to-expert” dualism highlights the developmental transition (common to all human skill learning) from slow, deliberate, step-by-step, unskilled performance to rapid, automatic, one-step (i.e., unitized) skilled processing. And because this broader dualism applies to *all* words in *all* orthographies, it seems a useful platform for developing a universal theory of learning to read.

### Conflict of interest statement

The author declares that the research was conducted in the absence of any commercial or financial relationships that could be construed as a potential conflict of interest.

## Footnotes

^1^This is by no means the first time an alphabetic writing system has been taught syllabically (see, for example, Cardoso-Martins, 1991; Liow Rickard and Lee, 2004). It is worth noting that Noah Webster's “blue-back speller” (first published in 1785) was also a syllable-based method of teaching English.

^2^I gloss over deep and unresolved issues regarding the linguistic and psycholinguistic status of morphemes and words, how these units might change in the course of literacy development, and how they are represented in diverse orthographies.
